# Azole-Resistant *Aspergillus fumigatus* Among Danish Cystic Fibrosis Patients: Increasing Prevalence and Dominance of TR_34_/L98H

**DOI:** 10.3389/fmicb.2020.01850

**Published:** 2020-08-13

**Authors:** Malene Risum, Rasmus Krøger Hare, Jan Berg Gertsen, Lise Kristensen, Helle Krogh Johansen, Jannik Helweg-Larsen, Nissrine Abou-Chakra, Tacjana Pressler, Marianne Skov, Søren Jensen-Fangel, Maiken Cavling Arendrup

**Affiliations:** ^1^Unit of Mycology, Statens Serum Institut, Copenhagen, Denmark; ^2^Department of Clinical Microbiology, Aarhus University Hospital, Aarhus, Denmark; ^3^Department of Clinical Microbiology, Rigshospitalet, Copenhagen, Denmark; ^4^Department of Clinical Medicine, University of Copenhagen, Copenhagen, Denmark; ^5^Department of Infectious Diseases, Rigshospitalet, Copenhagen, Denmark; ^6^Cystic Fibrosis Center Copenhagen, Department of Pediatrics and Infectious Diseases, Rigshospitalet, Copenhagen, Denmark; ^7^Department of Infectious Diseases, Aarhus University Hospital, Aarhus, Denmark

**Keywords:** *Aspergillus*, cystic fibrosis, azoles, resistance, mutation

## Abstract

Azole-resistant (azole-R) *Aspergillus* is an increasing challenge worldwide. Patients with cystic fibrosis (CF) are at risk of *Aspergillus* colonization and disease due to a favorable lung environment for microorganisms. We performed a nationwide study in 2018 of azole-non-susceptible *Aspergillus* in CF patients and compared with data from two prior studies. All airway samples with mold isolates from patients monitored at the two CF centers in Denmark (RH, Jan–Sept and AUH, Jan–Jun) were included. Classical species identification (morphology and thermo-tolerance) was performed and MALDI-TOF/β-tubulin sequencing was performed if needed. Susceptibility was determined using EUCAST E.Def 10.1, and E.Def 9.3.2. *cyp51A* sequencing and STR*Af* genotyping were performed for azole-non-susceptible isolates and relevant sequential isolates. In total, 340 mold isolates from 159 CF patients were obtained. The most frequent species were *Aspergillus fumigatus* (266/340, 78.2%) and *Aspergillus terreus* (26/340, 7.6%). Azole-R *A. fumigatus* was cultured from 7.3% (10/137) of patients, including 9.5% (9/95) of patients at RH and 2.4% at AUH (1/42), respectively. In a 10-year perspective, azole-non-susceptibility increased numerically among patients at RH (10.5% in 2018 vs 4.5% in 2007–2009). Cyp51A resistance mechanisms were found in nine azole-R *A. fumigatus* from eight CF patients. Five were of environmental origin (TR_34_/L98H), three were human medicine-driven (two M220K and one M220R), and one was novel (TR_34_^3^/L98H) and found in a patient who also harbored a TR_34_/L98H isolate. STR*Af* genotyping identified 27 unique genotypes among 45 isolates and ≥2 genotypes in 8 of 12 patients. This included one patient carrying two unique TR_34_/L98H isolates, a rare phenomenon. Genotyping of sequential TR_34_^3^/L98H and TR_34_/L98H isolates from the same patient showed only minor differences in 1/9 markers. Finally, azole-R *A. terreus* was found in three patients including two with Cyp51A alterations (M217I and G51A, respectively). Azole-R *A. fumigatus* is increasing among CF patients in Denmark with the environmentally associated resistance TR_34_/L98H mechanism being dominant. Mixed infections (wildtype/non-wildtype and several non-wildtypes) and a case of potential additional tandem repeat acquisition *in vivo* were found. However, similar genotypes were identified from another patient (and outside this study), potentially suggesting a predominant TR_34_/L98H clone in DK. These findings suggest an increasing prevalence and complexity of azole resistance in *A. fumigatus*.

## Introduction

Azole resistance (Azole-R) in *Aspergillus fumigatus* is an increasing problem and complicates patient management (Lestrade et al., 2019). Azole-R *A. fumigatus* in patients with cystic fibrosis (CF) has been reported in several studies (Mortensen et al., 2011a; Burgel et al., 2012; Morio et al., 2012; Bader et al., 2013; Fischer et al., 2014; Stevens et al., 2016; Prigitano et al., 2017; Abdolrasouli et al., 2018; Guegan et al., 2018; Güngör et al., 2018; Seufert et al., 2018; Engel et al., 2019; Lavergne et al., 2019; Table 1). Danish CF patients are followed up monthly at the two specialized CF clinics at Copenhagen University Hospital, Rigshospitalet (RH) and at Aarhus University Hospital (AUH). From the majority of these patients, airway samples are obtained monthly. We have previously studied azole-R *A. fumigatus* in the Copenhagen CF cohort (Mortensen et al., 2011a, b). The first study included isolates from Jan to March (Q1) 2007 (Mortensen et al., 2011b) and the second study isolates from July to Dec (Q3-4) of 2007 and of 2009 (Mortensen et al., 2011a). These studies documented an overall azole-non-susceptibility rate of 1.6 and of 4.5%, respectively. A lower but increasing rate from 1.8 to 3.8% of azole-R *A. fumigatus* among clinical samples (from CF as well as non-CF patients in Denmark) was found in a subsequent reference laboratory-based study, from the years 2010 to 2014 (Jensen et al., 2016). But the epidemiology of azole-R *A. fumigatus* including that specifically associated with environmental origin has not been systematically studied over a longer time in our country. Due to the increasing international and political concern related to the link between environmental azole fungicide use and azole resistance in *A. fumigatus*, we systematically investigated the azole resistance rate in the Danish CF population in 2018 and compared it to our previous data (Mortensen et al., 2011a).

**TABLE 1:** Review of published studies on azole resistance in patients with cystic fibrosis.

Study and year of publication	Country	Azole resistance rate at patient level	No. CF patients with *A. fumigatus*	No. *A. fumigatus* isolates in CF patients	Reported number of CF patients with *A. fumigatus* harboring a mutation of environmental origin	Reported number of CF patients with *A. fumigatus* harboring a mutation of non-environmental origin
Amorim et al., 2010	Portugal	0%*	11	159	None	None
Mortensen et al., 2011a	Denmark	4.5%	133	413	2/133 (1.5%) with TR_34_/L98H	4/133 (3%) one M220K, one Y4131C, one M220I and one non-*CYP51A* mutation
Burgel et al., 2012	France	4.6%	131	285	2/131 (1.5%) with TR_34_/L98H	4/131 (3.1%) one G54E, one M220I, one M220R and one non-*CYP51A* mutation
Morio et al., 2012	France	8%	50	85	3/50 (6%) with TR_34_/L98H	2/50 (4%) one M220T and one with TR_34_/L98H, G54R and M220T
Bader et al., 2013	Germany	5.5%**	na for cystic fibrosis	163	Three isolates with TR_34_/L98H***	One isolate with M220I, one isolate withF219C and four isolates with non-*CYP51A* mutations were also detected***
Fischer et al., 2014	Germany	3.4%	119	526	3/119 (2.5%). Three patients with TR_34_/L98H of which one with both TR_34_/L98H and TR_46_/Y121F/T289A	1/119 (8.4%) with M220L
Garczewska et al., 2016	Poland	0%	na for *A. fumigatus*	67	None	None
Stevens et al., 2016	United States	7%**	28	30	na	na
Prigitano et al., 2017	Italy	0% and 8.2% at the two centers	220	423	7/220 (3.2%) with TR_34_/L98H	1/220 (0.5%) with F219I
Abdolrasouli et al., 2018	United Kingdom	13.3% overall and 16.2% in CF patients specifically	74	N/A for CF patients	5/74 (6.8%) with TR_34_/L98H	None
Güngör et al., 2018	Turkey	16.7%	6	31	None	1/6 (16.7%) non-*CYP51A* mutation
Guegan et al., 2018	France	15.2%	33	42	1/33 (3%) with TR_34_/L98H	4/33 (12.1%) one M220K, one G54R and two non-*CYP51A* mutations
Seufert et al., 2018	Germany	5.3%	961	2888	89 isolates TR_34_/L98H and one isolate with TR_46_/Y121F/T289A***	Seven G54E, one G54R, one F219S and two non-*CYP51A* mutations***
Engel et al., 2019	Netherlands	7.1%****	558	2652	5.2% with TR_34_/L98H and 1.4% with TR_46_/T289A/Y121F****	0.2% with M220 and 0.2% with G54W****
Lavergne et al., 2019	France	6.8%	88	126	4/88 (4.5%). Two with TR_34_/L98H, one with TR_34_/L98H/S297T/F495I and one with TR_46_/Y121F/T289A	2/88 (2.3%) one F46Y/M172V/N248T/D255E/E427K and one non-*CYP51A* mutation

CF: Cystic fibrosis. A. fumigatus: Aspergillus fumigatus na: Not available. *One patient had one isolate with MIC outside of the epidemiological cut-off of 0.25 μg/mL for posaconazole. **Azole resistance at isolate level in CF patients. ***The number of mutations per patient was not reported. ****Mean per year.

CF is the most common autosomal recessive disease in Caucasians (Felton and Simmonds, 2014). Mutations in the CFTR (cystic fibrosis transmembrane regulator gene) affects the chloride transportation causing dysregulated fluid transport in the epithelial cells of multiple organs (Felton and Simmonds, 2014). Clinically, CF disease is dominated by infectious pulmonary complications (Felton and Simmonds, 2014). The respiratory tract is often colonized with molds especially *A. fumigatus*, which is found in 16 to 56.7% of airway samples (Pihet et al., 2009). *Aspergillus* may cause a diversity of manifestations ranging from asymptomatic colonization to serological sensitization, allergic bronchopulmonary aspergillosis (ABPA), *Aspergillus* bronchitis, and aspergilloma in CF patients (Felton and Simmonds, 2014). The most common is ABPA (Pihet et al., 2009), which occurs in approximately 10% of CF patients (Burgel et al., 2016; Carsin et al., 2017) and is the cause of hypersensitivity response to *Aspergillus* antigens (Williams et al., 2016). Azoles are the cornerstone in the management of CF patients with *Aspergillus* disease requiring antifungal therapy. Itraconazole is the first choice as an antifungal drug in the treatment of ABPA to reduce the burden of *A. fumigatus* and minimize use of corticosteroids (Patterson et al., 2016). Posaconazole is used as salvage therapy in ABPA or bronchitis (Skov et al., 2017; Periselneris et al., 2019), whereas voriconazole or isavuconazole are first-line agents (Maertens et al., 2016; Patterson et al., 2016) in the rare event of invasive aspergillosis (Burgel et al., 2016; Skov et al., 2017; Hamprecht et al., 2018).

Azoles target and inhibit the lanosterol 14-α-demethylase enzyme (Cyp51A) encoded by the *cyp51A* gene and thereby inhibit the ergosterol synthesis (Stensvold et al., 2012). Patients with recurrent or long-term need for azole therapy are at risk for azole-R *Aspergillus* due to selection of resistance during exposure to medical azoles (Hamprecht et al., 2018). Azole-R in *A. fumigatus* also occurs in patients with no prior azole therapy, caused by the inhalation of resistant mutant spores from the environment presumably selected due to azole fungicide use for plant and material protection (Astvad et al., 2014; Hagiwara et al., 2016). Well-known mechanisms behind azole resistance are target gene mutations in *cyp51A*. Two common resistance mechanisms, TR_34_/L98H and TR_46_/Y121F/T289A, are considered to be of environmental origin (Stensvold et al., 2012). These “environmental” mechanisms have previously been found in the Danish environment (Mortensen et al., 2010; Risum et al., 2019) and in clinical samples (Mortensen et al., 2011a; Astvad et al., 2014). Furthermore, target gene upregulation, efflux, and HapE (Camps et al., 2012) and Hmg1 (Rybak et al., 2019) alterations have been documented as underlying mechanisms of azole resistance in selected isolates.

In this study, we investigated the azole-R rate in a 10-y perspective and dissected underlying molecular resistance mechanisms and genotypes in *Aspergillus* in CF patients followed up at the two Danish CF centers that serve the entire country.

## Materials and Methods

The two CF centers RH and AUH follow all the Danish CF patients. A total of 522 (320 and 202 adult and children) with CF were followed up at RH and AUH, respectively, in 2018. All positive cultures with mold from airway samples from the Danish CF population were included during a 6-month (Jan–June 2018) and a 9-month (Jan–Sept 2018) period, respectively, from AUH and RH. Primary culture was performed using Sabouraud glucose agar [SSI Diagnostika, Hillerød, Denmark (RH) and bioMérieux, Marcy l’Etoile, France (AUH), respectively]. Agar plates were incubated at 35–37°C and examined for 5 days (RH) and 2 days (AUH). Exclusion criteria were identical to our previous study (Mortensen et al., 2011a). In detail, repeat isolates from the same patient were excluded when found ≤30 days apart and confirmed as same species and with same susceptibility classification.

Identification was done to the *Aspergillus* species complex level using classical techniques, including thermo-tolerance test for *A. fumigatus* specifically, followed up by MALDI-TOF applying the Mass Spectrometry Identification database (Normand et al., 2017; Imbert et al., 2019) and β-tubulin sequencing (Glass and Donaldson, 1995) when necessary.

The EUCAST E.Def 10.1 method (Arendrup et al., 2017) was used for azole-R screening for *A. fumigatus*, and EUCAST E.Def 9.3.1 susceptibility testing (Arendrup et al., 2016) was performed for amphotericin B for the majority of the isolates and for itraconazole, posaconazole, and voriconazole for screening-positive *A. fumigatus* isolates and *Aspergillus* species other than *A. fumigatus*. Isolates with azole MIC(s) above the ECOFF(s) underwent *cyp51A* sequencing as previously described (Mortensen et al., 2011a). EUCAST clinical breakpoints v 9.0 were adopted for susceptibility classification into susceptible, non-susceptible (intermediate and resistant), and azole-R (Arendrup et al., 2013). For species and agents without breakpoints, EUCAST ECOFFs were adopted and non-wildtype isolates were regarded resistant. Sequential isolates from all patients harboring resistant *A. fumigatus* underwent STRA*f* genotyping (De Valk et al., 2005).

Results were compared to our previous Danish studies on azole-R in CF patients followed up at RH allowing a 10-year perspective (Mortensen et al., 2011a). Comparison of groups was performed with a contingency chi-square test using GraphPad Prism version 8.0.2.

(Preliminary results from RH have been presented at the European Congress on Clinical Microbiology and Infectious Diseases in 2019).

## Results

In total, 340 unique mold isolates from 159 CF patients were obtained, of which 240 isolates were derived from 110 CF patients at RH (2.2 isolates per patient) and 100 isolates from 49 CF patients at AUH (2.0 isolates per patient). The median age was 30 years (6–68 years) at RH and 22 years (6–50 years) at AUH among patients with a mold isolate.

**FIGURE 1:**
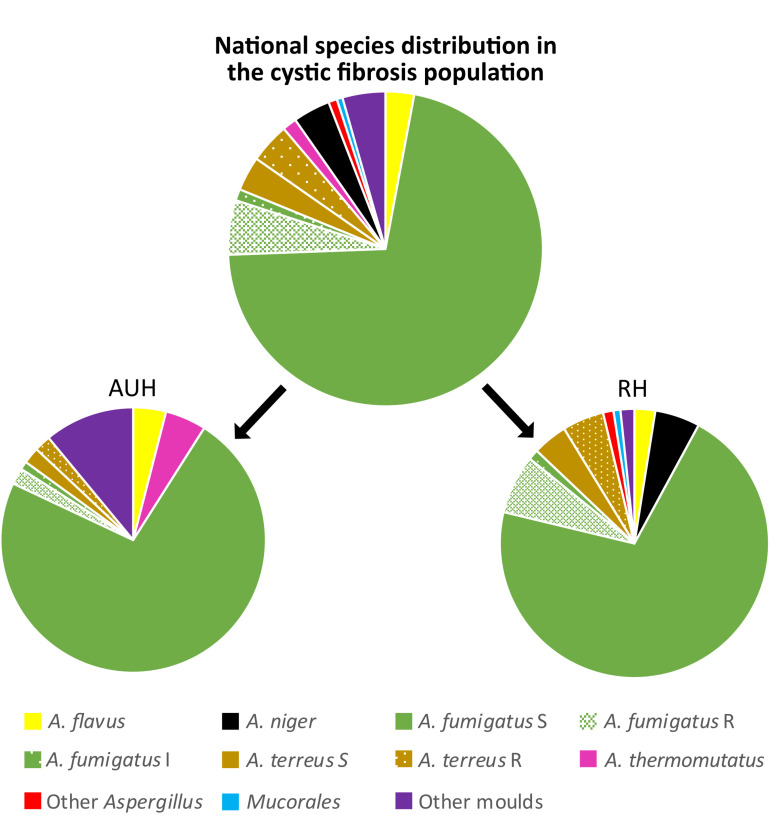
Species distribution at the cystic fibrosis population gathered and at the two centers separately. S, I, and R indicate the susceptibility for azoles. S: susceptible, I: intermediate, and R: resistant. Aarhus (AUH): Other molds: *Scedosporium* spp. (*n* = 8), *Exophiala* spp. (*n* = 2), *Penicillium* spp. (*n* = 1). Rigshospitalet (RH): Other *Aspergillus*: *Aspergillus nidulans* (*n* = 2) and *Aspergillus sydowii* (*n* = 1), *Mucorales: Rhiomucor pusillus* (*n* = 1) *and Zygomycetes* spp. (*n* = 1). Other molds: *Scedosporium* spp. (*n* = 3), and *Rasamsonia* spp. (*n* = 1).

Overall, *A. fumigatus* was the most frequently isolated species (266/340 isolates, 78.2%), detected in 137/159 (86.2%) of the CF patients followed up by *A. terreus* species complex (26/340 isolates, 7.6%) in 10/159 patients (6.3%) and *A. niger* complex isolates (13/340 isolates, 3.8%) in 13/159 (8.2%) patients (Figure 1).

Azole non-susceptible *A. fumigatus* was found in 12/137 (8.8%) and Azole-R *A. fumigatus* in 10/137 (7.3%) of all CF patients (Table 2). Eight patients harbored *A. fumigatus* with Cyp51A alterations, including one patient (RH-5) with two isolates with different Cyp51A alterations. Overall, Cyp51A alterations were thus found in 81.8% (9/11) azole-R *A. fumigatus* of which 45.5% (5/11) were of environmental origin (Table 2). Three *A. fumigatus* isolates were categorized as intermediate to one or several azoles, none of which harbored *cyp51A* mutations.

**TABLE 2:** Cyp51A profile for 18 *Aspergillus fumigatus* and *Aspergillus terreus* isolates with decreased susceptibility from 15 CF patients at the two referral centers in Denmark.

Species/patient ID^a^	Susceptibility classification^b^	Cyp51A profile
***A. fumigatus***		
AUH-1	Intermediate	Wildtype
AUH-2	Resistant	TR_34_/L98H
RH-1	Intermediate	Wildtype
RH-2	Intermediate	Wildtype
	Resistant	Wildtype
RH-3	Resistant	Wildtype
RH-4	Resistant	TR_34_/L98H/S297T/F495I
RH-5	Resistant	TR_34_^3^/L98H
	Resistant	TR_34_/L98H
RH-6	Resistant	TR_34_/L98H
RH-7	Resistant	TR_34_/L98H
RH-8	Resistant	M220R
RH-9	Resistant	M220K
RH-10	Resistant	M220K
***A. terreus***		
AUH-3	Resistant	
RH-11	Resistant	M217I
	Intermediate	Y491H
RH-12	Resistant	G51A

*^a^AUH, Aarhus University Hospital, RH, Rigshospitalet. ^b^According to EUCAST clinical breakpoints for antifungals v 9.0, for A. fumigatus an isolate was considered resistant when MIC for ITR was >2 mg/L, POS > 0.25 mg/L and VOR > 2 mg/L. It was considered intermediate when MIC for itraconazole was 2 mg/L, posaconazole 0.25 mg/L and voriconazole 2 mg/L.*

*A. fumigatus* was equally common among mold-colonized patients at the two centers [42/49 (85.7%) and 95/110 (86.4%)], but the proportion of patients with non-susceptible *A. fumigatus* isolates differed. At AUH, non-susceptible *A. fumigatus* isolates were observed in 2/42 (4.8%) patients (Table 2). At RH, 10/95 (10.5%) patients carried non-susceptible *A. fumigatus.* Nine of these patients (9.5%) carried 10 azole-R *A. fumigatus.* Eight patients had a *cyp51A* mutation (7.4% of patients), five of which with tandem repeats (TR_34_/L98H or TR_34_^3^/L98H) and three with alterations affecting the M220 codon (Table 2). Among the 10 patients with resistant *A. fumigatus*, four patients had only resistant isolates obtained in the study period. These included the resistance mechanisms TR_34_/L98H, TR_34_^3^/L98H, and wild-type. Alternating resistant and susceptible *A. fumigatus* isolates were found in six patients with the resistance mechanisms M220K, TR_34_/L98H, TR_34_/L98H/S297T/F495I, M220R, and wild-type (M220K two patients and one each).

STR*Af* genotyping identified 27 unique STR*Af* genotypes among the 45 *A. fumigatus* isolates from the 12 patients with azole non-susceptible *A. fumigatus* (Table 3). Eight patients harbored isolates with more than one genotype. Three of these patients, carried isolates that differed only in a single marker (RH-5, RH-7, and RH-8), and six patients carried isolates that were clearly unrelated (including two patients with both related and unrelated genotypes RH-7). Thus, patient RH-5 had six isolates with 8/9 identical STR*Af* markers, while marker 3A ranged from 95 repeats (TR_34_/L98H) to 96–101 repeats (five TR_34_^3^/L98H) (Table 3). In contrast, Patient AUH-2 harbored two TR_34_/L98H isolates but with clearly different STR*Af* profiles. Patient RH-2 harbored 10 *A. fumigatus* with three different genotypes, while patient RH-8 had four isolates with 8/9 identical markers and a fifth isolate with 7/9 identical markers. Isolates that shared 8–9 markers were also found across patients. Thus, patient RH-7 had two TR_34_/L98H isolates with identical STR*Af* profiles as two TR_34_^3^/L98H isolates from RH-5.

**TABLE 3:** Detailed susceptibility pattern and STRAf genotyping of all *Aspergillus fumigatus* isolates from patients harboring isolates with decreased azole susceptibility.

Patient ID	MIC (mg/L)			Cyp51A profile	Sample date	Genotype								
	ITR	POS	VOR			2A	2B	2C	3A	3B	3C	4A	4B	4C
AUH-1	1	0.25	2	Wild-type	15-05-2018	26	20	8	36	9	6	8	10	20
AUH-2	>16	1	4	TR_34_/L98H	10-01-2018	10	20	10	29	9	6	8	10	19
	>16	1	8	TR_34_/L98H	04-04-2018	23	21	16	65	9	9	10	24	21
	>16^*b*^	0.5	4	TR_34_/L98H	03-05-2018	23	21	16	65	9	9	10	24	21
RH-1	1	0.25	2	Wild-type	22-06-2018	23	23	15	23	11	19	13	9	5
	2	0.25	2	Wild-type	17-08-2018	23	23	15	23	11	19	13	9	5
RH-2	>16	2	2	Wild-type	08-02-2018	17	12	14	34	65	12	8	9	5
	S	S	S	NA, S	15-03-2018	18	21	19	22	11	28	10	8	8
	2	0.25	2	Wild-type	19-04-2018	17	12	14	34	65	12	8	9	5
	S	S	S	NA, S	19-04-2018	18	21	19	22	11	28	10	8	8
	S	S	S	NA, S	18-05-2018	18	21	19	22	11	28	10	8	8
	2	1	1	Wild-type	07-06-2018	17	12	14	34	65	12	8	9	5
	S	S	S	NA, S	29-06-2018	18	21	19	22	11	28	10	8	8
	S	S	S	NA, S	16-08-2018	17	12	14	34	65	12	8	9	5
	S	S	S	NA, S	16-08-2018	18	21	19	22	11	28	10	8	8
	0.5	0.125	2	Wild-type	20-09-2018	18	21	19	22	11	28	10	8	8
	0.5	0.125	3	Wild-type	20-09-2018	19	24	20	36	11	17	16	11	8
RH-3	8	0.25	2	Wild-type	11-05-2018	21	25	18	27	12	6	21	10	8
RH-4	>16	4	8	TR_34_/L98H/S297T/F495I	26-04-2018	14	20	8	40	9	10	8	10	20
	>16	2	4	TR_34_/L98H/S297T/F495I	20-07-2018	14	20	8	40	9	10	8	10	20
	0.25	0.06	0.5	Wild-type	23-08-2018	14	20	8	36	9	9	8	10	20
RH-5	NA	NA	NA	Wild-type	11-07-2009	25	20	8	10	10	21	9	10	5
	S	S	S	NA, S	27-01-2010	NA	NA	NA	NA	NA	NA	NA	NA	NA
	>16	1	4	TR_34_^3^/L98H	25-05-2017	23	21	16	97	9	6	5	9	10
	>16	1	8	TR_34_^3^/L98H	31-05-2017	23	21	16	96	9	6	5	9	10
	>16	1	4	TR_34_^3^/L98H	16-01-2018	23	21	16	98	9	6	5	9	10
	>16	1	4	TR_34_/L98H	13-02-2018	23	21	16	95	9	6	5	9	10
	>16	1	4	TR_34_^3^/L98H	13-02-2018	23	21	16	99	9	6	5	9	10
	>16	1	8	TR_34_^3^/L98H	01-03-2018	23	21	16	101	9	6	5	9	10
RH-6	>16	1	8	TR_34_/L98H	21-06-2018	14	20	17	31	11	9	8	14	20
	>16	1	8	TR_34_/L98H	01-10-2018	14	20	17	31	11	9	8	14	20
RH-7	0.25	0.06	0.5	NA, S	17-09-2018	14	18	12	34	9	7	8	10	21
	0.125	0.06	0.5	NA, S	06-09-2018	18	20	9	26	10	6	8	10	5
	8	0.5	4	TR_34_/L98H	10-05-2018	23	21	16	97	9	6	5	9	10
	>16	0.5	4	TR_34_/L98H	05-07-2018	23	21	16	99	9	6	5	9	10
RH-8	>16	>4	2	M220R	06-02-2018	23	23	15	40	11	46	10	26	8
	>16	4	1	NA, R	27-03-2018	23	23	15	40	11	46	10	26	8
	>16	>4	2	Mixed, M220R and Wild-type	03-04-2018	23	23	15	40	11	46	10	26	8
	>16	>4	2	Mixed, M220R and Wild-type	03-04-2018	23	23	15	41	11	46	10	26	8
	>16	2	2	Wild-type	04-09-2018	23	23	15	40	11	45	10	26	8
	0.5	0.25	1	Wild-type	20-09-2018	23	23	15	37	11	42	10	26	8
RH-9	>16	1	1	M220K	07-03-2018	18	19	11	27	10	37	20	11	5
	0.5	0.125	0.5	NA, S	27-08-2018	18	28	8	29	11	17	8	8	5
RH-10	>16	2	1	M220K	04-03-2018	11	12	11	17	9	15	8	8	5
	S	S	S	NA, S	03-05-2018	18	23	26	22	11	25	22	10	8

*^a^AUH, Aarhus University Hospital, RH, Rigshospitalet. ITR, Itraconazole, POS, Posaconazole, VOR, Voriconazole, S, Susceptible, R, Resistant, NA, Not analyzed. ^b^Duplicate isolates and isolates outside the study period are shown in gray.*

Azole resistance was also detected in other *Aspergillus* species. At AUH, such isolates were found in 3/49 (6.1%) patients including two patients with *A. thermomutatus*, and one patient with a voriconazole-resistant *A. terreus* isolate with a wild-type *cyp51A* (Table 2). At RH, two out of eight CF patients with *A. terreus* (1.8% of CF patients at RH) had non-susceptible isolates. One patient (RH-11) had both resistant and intermediate isolates recovered, which had Cyp51A amino acid substitutions M217I and Y491H, respectively. The other patient had a resistant *A. terreus* with a G51A mutation (RH-12, Table 2). MIC distributions for all *Aspergillus* isolates with reduced azole susceptibility at the two centers are shown in Supplementary Table 1.

Finally, we investigated potential changes in non-susceptibility and Cyp51A alteration rates in a 10-year perspective by comparison with data from the period 2007-2009 for the RH CF cohort (Mortensen et al., 2011a, b). Non-susceptible *A. fumigatus* was observed in 1/61 (1.6%) patients in Q1-2007, 6/133 patients in 2007–9 (4.5%) compared to 10/95 (10.5%) in 2018 (*P* = 0.047). Similarly, the proportion of isolates with Cyp51 alterations in Q1-2007, Q3-4-2007/9, and 2018 increased: 1.6% (95% CI: 0.1–8.7%), 3.8% (95% CI: 1.6–8.5%) and 7.4% (95% CI: 3.6–14.4%), respectively. Finally, the number of patients with resistant isolates with a tandem repeat, specifically, increased during the three study periods: 0% (95% CI: 0–5.9%), 1.5% (95% CI: 0.3–5.3%), and 4.2% (1.6–10.3%), as well as the proportion of isolates with target gene mutations associated with long-term azole treatment: 1.6% (95% CI: 0.8%-8.7%), 2.3% (95% CI: 0.6%-6.4%), and 3.2% (95% CI: 0.9%-8.9%) in Q1-2007, Q3-4-2007/9, and 2018, respectively.

## Discussion

We report detailed and nationwide data on azole non-susceptibility and mold species distribution in respiratory isolates from Danish CF patients. Azole-R *A. fumigatus* with environmental origin was dominating and found at both centers suggesting a wide geographic distribution of TR_34_/L98H in Denmark. However, although the proportions of patients with *A. fumigatus* (85.7 and 86.3%, respectively) were similar at the two centers, an almost four-fold higher rate of azole-resistant *A. fumigatus* was observed at RH compared to AUH. Moreover, the resistance pattern was more diverse at RH and included both the resistance deriving from the environment and resistance mutations associated with azole treatment selection. Unfortunately, data on azole use in these patients could not be retrieved. However, it is most likely that the differences in azole-R between the two centers may be related to different prescription practices with a more extensive and longer duration of azole treatment at RH, in part due to an overall higher age and number of patients with chronic aspergillus bronchitis at RH. This is supported by the observation that human-driven target gene mutations were more common in patients at RH than at AUH.

At RH, *cyp51A* mutations of environmental origin accounted for half of the detected resistance, and the TR_34_/L98H rate has doubled over the past decade since the first detection in Q3-4 of 2007–9 (Mortensen et al., 2011a). Additionally, a subsequent laboratory study of *A. fumigatus* isolates received at the national reference center reported an increase during 2010–2014 (Jensen et al., 2016). Taken together, these studies suggest that TR_34_/L98H has gradually become more prevalent in Denmark since 2007 despite the fact that two of three environmental sampling studies in Denmark failed to detect TR_34_/L98H and TR_46_/Y121F/T289A in soil and air samples (Astvad et al., 2014; Jensen et al., 2016). This suggests either significant fluctuations in the number of resistant spores in the environment or that even low levels of resistant *A. fumigatus* can contribute to resistant infections in a predisposed lung environment.

Our observations of alternating or mixed resistant and susceptible isolates recovered from the same patient is a well-known phenomenon and highlight that a single sample may not be a representation for the entire lung flora (van Leer-Buter et al., 2007; Astvad et al., 2014). Not only may different phenotypes dominate in different lung sections but mixed *A. fumigatus* strains are also very challenging to identify and separate unless molecular analyses are performed. Of note, two TR_34_/L98H isolates with different and unique STR*Af* genotypes among our collection were recovered from patient AUH-2, a case which we have not previously seen in DK.

Of particular interest, patient RH-5 harbored five pan-azole-resistant isolates with a novel TR_34_^3^/L98H resistance mechanism, which to our knowledge has not previously been found in clinical specimens. However, exposure *in vitro* of *A. fumigatus* conidia already containing a 34-bp insertion in the *cyp51A*-gene promoter to 8 mg/L of tebuconazole resulted in one clone with a 34-bp triplicate repeat (Snelders et al., 2012). In addition, the TR_46_/Y121F/T289A has also been found with additional 46-bp repeats in the promoter region in compost as well during sexual mating in *in vitro* studies (Zhang et al., 2017). The question remains whether this TR_34_^3^/L98H resistance variant is novel in the environment and thus acquired *de novo*, as suggested by being isolated first, or whether the TR_34_/L98H was in fact first (but undiscovered in the first three specimens) and the additional TR_34_ repeat acquired *in vivo*. The STR*Af* profiles suggest that the five TR_34_^3^/L98H isolates are isogenic with a classical example of microevolution. It is noteworthy that the TR_34_/L98H isolate from this patient shared 8/9 markers and had 95 repeats at marker 3A while an increasing number of repeats (96–101) were seen in the TR_34_^3^/L98H isolates over time. Increasing repeat numbers have previously been found over time *in vitro* and *in vivo* (Mortensen et al., 2011a; De Groot and Meis, 2019). Furthermore, the TR_34_/L98H strain was discovered in a sample mixed with the TR_34_^3^/L98H strain and had a white and slow-growing phenotype, which could help explain why it could potentially have been overlooked in earlier samples. Indeed, the *in vivo* acquisition of a tandem repeat in the promotor region has been reported from our group, where a 120-basepair tandem repeat evolved in a patient during azole therapy, supported by whole-genome sequencing (WGS) (Hare et al., 2019). It has also been suggested that the TR_34_ helps compensate for loss of fitness associated with the L98H change (Verweij et al., 2016) and thus the additional TR_34_ could potentially further improve fitness and outgrow the TR_34_/L98H, which in this patient appeared with a weaker phenotype (Verweij et al., 2016). A third hypothesis could be that this is a random coincidence of similar STR*Af* genotypes. Indeed, the finding of two TR_34_/L98H isolates from another patient (RH-7) displaying identical STR*Af* profiles as two TR_34_^3^/L98H isolates was surprising and further complicates the interpretation. One concern would be lab contamination, but since the two RH-7 isolates were received months apart and with different *cyp51A* profiles, this seems unlikely. A final, and worrying, theory is that we may have encountered a dominating TR_34_/L98H clone in DK similar to the study from India (Chowdhary et al., 2012). Indeed, outside the study period we have encountered a total of 20 isolates from 12 different patients from all around DK and also in two air samples sharing the same 8/9 STR*Af* markers, exclusively differing in marker 3A, ranging from 35 to more than 130 repeats. The high variation in 3A (in our two patients) indicates a highly mutagenic strain type, which may help explain the rare development of the TR_34_^3^/L98H variant. Further studies including WGS are desirable to further explore the origin of this novel resistance mechanism as well as the potentially novel dominating genotype.

Two CF patients had resistant *A. fumigatus* with wild-type *cyp51A*, as reported in other CF studies (Mortensen et al., 2011a; Burgel et al., 2012; Guegan et al., 2018; Seufert et al., 2018; Lavergne et al., 2019) at similar rates (Mortensen et al., 2011a; Burgel et al., 2012; Lavergne et al., 2019) as well as in patients with chronic pulmonary aspergillosis (Howard et al., 2013). Phenotypic susceptibility testing therefore remains crucial because molecular detection of resistance mechanism enables the detection of resistance, but not susceptibility. Moreover, alternating findings of susceptible and non-susceptible isolates in the same patient demonstrate the need of repeated sampling and susceptibility testing of several colonies when present in patients requiring azole therapy, as recommended in current guidelines (Ullmann et al., 2018; Guinea et al., 2019).

Azole-R *A. terreus* constitutes a significant challenge since *A. terreus* has intrinsic reduced susceptibility to amphotericin B, rendering it multidrug-resistant (Arendrup et al., 2012; Zoran et al., 2018; Rivero-Menendez et al., 2019). The present finding of *A. terreus* of 6.3% (10/159) nationally at patient level is quite high compared to previous studies (Mortensen et al., 2011a; Fischer et al., 2014; Engel et al., 2019), reporting 1.9% (Mortensen et al., 2011a) and 2.4% *A. terreus* at the isolate level (Fischer et al., 2014) and 3.9% of all *Aspergillus* spp. (Engel et al., 2019). Whereas M217I has been reported previously (Rivero-Menendez et al., 2019), G51A is, to our knowledge, novel. We also detected *A. thermomutatus*, another inherently voriconazole-resistant *Aspergillus* spp., at AUH. This species has been detected in one CF patient at RH previously (Mortensen et al., 2011a) illustrating that resistant *Aspergillus* infection is not limited to *A. fumigatus* in this setting.

When we compare the present study’s result to others, the current overall azole-R rate of 7.3% in the total CF population corresponded well with CF studies from other European countries [France, Germany, the Netherlands (Bader et al., 2013; Seufert et al., 2018; Engel et al., 2019; Lavergne et al., 2019), and the United States (Table 1)] (Stevens et al., 2016). Internationally, published azole resistance rates have varied greatly. No azole resistance was reported from a Portuguese center (Amorim et al., 2010), in one of two Italian centers (Prigitano et al., 2017), and in five out of 12 German centers (Seufert et al., 2018; Table 3). In contrast, Abdolrasouli et al. found a concerning high resistance rate of 16.2% in CF patients specifically, which could be reflected upon the patient group at a cardiothoracic center in United Kingdom following up CF patients (Abdolrasouli et al., 2018). Guegan et al. also found a high azole-R rate of 15.2%, but in a limited CF population of 33 patients (Guegan et al., 2018; Table 1).

The major strength of the present study is the fact that it allowed a 10-year perspective on azole-resistant *Aspergillus* and a nationwide surveillance perspective of the current mold epidemiology in CF patients in Denmark. Since we also included *Aspergillus* species other than *A. fumigatus*, we also reported mutations in *A. terreus* and furthermore detailed information on STR*Af* genotyping in one patient with *A. fumigatus*. A limitation, however, is that we do not have information regarding the clinical relevance of the retrieved *A. fumigatus*, nor do we have any information regarding preceding antifungal use.

In conclusion, azole-R *Aspergillus* is increasing in proportion and complexity among Danish CF patients. The larger and increasing proportion involved resistant *A. fumigatus* of environmental origin, and novel genotypes in both *A. fumigatus* and *A. terreus* were found. Although the isolation of *Aspergillus* may reflect contamination or transient colonization and thus include patients in whom antifungal therapy is not indicated, the continuously emerging reports of azole-resistant *Aspergillus* is worrisome, and resistance remains a significant challenge. This is of concern as effective alternative treatments to azoles are lacking and as it suggests that azole-resistant *A. fumigatus* may also be an increasing challenge in other patient populations at risk for aspergillus disease.

## Data Availability Statement

The raw data can be provided from the corresponding author according to the Danish law.

## Ethics Statement

Ethical review and approval was not required for the study on human participants in accordance with the local legislation and institutional requirements. Written informed consent from the patients was not required to participate in this study in accordance with the national legislation and the institutional requirements.

## Author Contributions

MR, MA, and HJ designed the study. HJ, JG, and LK were responsible for primary cultures and isolation. MA, JG, and LK were responsible for the susceptibility testing. RH and NA-C performed the molecular analysis. MR performed the data management. MR and MA wrote and revised the manuscript after review from all co-authors. All authors contributed to the article and approved the submitted version.

## Conflict of Interest

MR has over the past 5 years received speaker honoraria from BMS and, unrestricted research and travel grants from Novartis. JG has, over the past 5 years, received travel grants from Gilead and speaker honoraria from Gilead and MSD. RH has received unrestricted research grants from Gilead and conference meeting grants from Gilead, MSD, Pfizer, and Astellas. MA has, over the past 5 years, received research grants/contract work (paid to the SSI) from Amplyx, Basilea, Cidara, F2G, Gilead, Novabiotics, Scynexis, and T2Biosystems and speaker honoraria (personal fee) from Astellas, Gilead, Novartis, MSD, and Seges. She is the current chairman of the EUCAST-AFST. The remaining authors declare that the research was conducted in the absence of any commercial or financial relationships that could be construed as a potential conflict of interest.
