# Supplementary material for: Azole-Resistant Aspergillus fumigatus Among Danish Cystic Fibrosis Patients: Increasing Prevalence and Dominance of TR34/L98H
Source: Front Microbiol. 2020 Aug 13;11:1850. doi: 10.3389/fmicb.2020.01850 (PMC7438406; doi:10.3389/fmicb.2020.01850)
Supplement: Supplementary file 1 [file Table_1.docx]

**Supplementary table 1.** Azole MIC distributions for isolates belonging to the three *Aspergillus* species for which acquired or intrinsic azole resistance was found. MICs classified as intermediate are indicated in bold and those resistant (or non-wildtype for species without breakpoints) in bold and underlined. *The four A. terreus isolates with itraconazole MIC = 1 mg/L are underlined to highlight that these isolates are non-wild type for itraconazole (ECOFF=0.5 mg/L) but categorised as susceptible (S ≤ 1 mg/L).

| **Species, azole and CF centre^a^** |  | **Number of isolates with the given MIC (mg/l)** | | | | | | | | | | | |
| --- | --- | --- | --- | --- | --- | --- | --- | --- | --- | --- | --- | --- | --- |
|  |  | **S** | **0.06** | **0.125** | **0.25** | **0.5** | **1** | **2** | **4** | **≥8** | **16** | **>16** | **Not done** |
| ***Aspergillus fumigatus*** |  |  |  |  |  |  |  |  |  |  |  |  |  |
| **Itraconazole** |  |  |  |  |  |  |  |  |  |  |  |  |  |
| AUH |  | 63 |  | 1 | 5 | 3 | 2 |  |  |  |  | **2** |  |
| RH |  | 132 |  | 1 | 19 | 15 | 4 | **3** |  | **2** |  | **14** |  |
| **Posaconazole** |  |  |  |  |  |  |  |  |  |  |  |  |  |
| AUH |  | 63 | 5 | 5 | **1** |  | **2** |  |  |  |  |  |  |
| RH |  | 134 | 10 | 23 | **6** | **3** | **6** | **4** | **2** | **2** |  |  |  |
| **Voriconazole** |  |  |  |  |  |  |  |  |  |  |  |  |  |
| AUH |  | 63 |  |  |  | 6 | 4 | **1** | **1** | **1** |  |  |  |
| RH |  | 132 |  |  |  | 25 | 16 | **9** | **5** | **3** |  |  |  |
| ***Aspergillus terreus*** |  |  |  |  |  |  |  |  |  |  |  |  |  |
| **Itraconazole** |  |  |  |  |  |  |  |  |  |  |  |  |  |
| AUH |  |  |  | 3 | 1 |  |  |  |  |  |  |  |  |
| RH |  |  | 3 | 2 | 4 |  | 4 | **3** | **1** |  |  | **5** |  |
| **Posaconazole** |  |  |  |  |  |  |  |  |  |  |  |  |  |
| AUH |  |  | 1 | 3 |  |  |  |  |  |  |  |  |  |
| RH |  |  | 4 | 4 | 3 | **9** | **2** |  |  |  |  |  |  |
| **Voriconazole** |  |  |  |  |  |  |  |  |  |  |  |  |  |
| AUH |  |  |  |  |  |  | 1 | 1 | **2** |  |  |  |  |
| RH |  |  |  |  |  | 2 | 8 | 5 | **6** | **1** |  |  |  |
| ***Aspergillus thermomutatus*** |  |  |  |  |  |  |  |  |  |  |  |  |  |
| **Itraconazol** |  |  |  |  |  |  |  |  |  |  |  | 5 |  |
| **Posaconazol** |  |  |  |  | 1 | 4 |  |  |  |  |  |  |  |
| **Voriconazol** |  |  |  |  |  |  |  |  | 3 | 2 |  |  |  |

^a^AUH: Aarhus University Hospital, RH: Rigshospitalet

*Aspergillus thermomutatus* was only detected at AUH

Grey indicates that the isolate was not tested at these concentrations
